# The frequency of psoriasis in Australian multiple sclerosis patients

**DOI:** 10.3389/fneur.2025.1663015

**Published:** 2025-10-02

**Authors:** Varitsara Mangkorntongsakul, Ariadna Fontes-Villalba, Turlough Montague, Olivia Charlton, Chun-Ting Justin Kwong, Helen Marie McGuire, Venkatesha Venkatesha, Geoffrey Herkes, Saxon Smith, John Parratt

**Affiliations:** ^1^Faculty of Medicine and Health, Northern Clinical School, The University of Sydney, Darlington, NSW, Australia; ^2^Department of Neurology, Royal North Shore Hospital, St. Leonards, NSW, Australia; ^3^Faculty of Medicine and Health, School of Medical Sciences, The University of Sydney, Sydney, NSW, Australia; ^4^Northern Sydney Local Health District Executive, Northern Sydney Local Health District, St. Leonards, NSW, Australia; ^5^Department of Dermatology, Sydney Adventist Hospital, Wahroonga, NSW, Australia; ^6^The Dermatology and Skin Cancer Centre, Gosford, Sydney, NSW, Australia; ^7^ANU Medical School, ANU College Health and Medicine, The Australian National University, Canberra, ACT, Australia

**Keywords:** Australian population, multiple sclerosis, psoriasis, neuroimmunology, epidemiology study, autoimmune diseases

## Abstract

**Background:**

Multiple Sclerosis (MS) is an immune-mediated, demyelinating disease of the central nervous system. Although psoriasis and psoriasiform dermatitis are reported in MS patients, the prevalence of the diseases is uncertain globally and unstudied in Australia. This study aims to determine the frequency of psoriasis in a clinic-based cohort of Australian MS patients.

**Methods:**

A survey was conducted on 204 consecutive MS patients aged 18 and over who attended a tertiary MS clinic in Northern Sydney from July 2018 to December 2022.

**Results:**

A total of 204 patients were examined, comprising 137 female (67.2%) and 67 male (32.8%). The mean age was 48.8 years (SD = 13.6). Psoriasis was identified in 13.7% (28/204; 95% CI: 9.63% to 19.20%).

**Discussion:**

The frequency of psoriasis in MS is high and may be underestimated, given that many more patients have symptoms without signs. This implies an immunopathological link between the two conditions and is worthy of further study.

## Introduction

Multiple Sclerosis (MS) is an immune-mediated, demyelinating disease of the central nervous system (1, 2). Although recent work and clinical data imply an increasing role for B-lymphocytes in the pathogenesis of MS, aberrant T-lymphocyte responses are likely necessary for the development of the disease and have been demonstrated among patients (3–5). Further, experimental models utilize myelin-specific T-lymphocytes (6, 7) to induce macrophage mediated, inflammatory demyelination and these changes recapitulate many elements of the human disease. To add complexity, the precise immunopathological mechanisms in individual MS patients may vary given the clinical and radiological heterogeneity of the disease, variance in the clinical course of MS and response to treatment (5, 8, 9).

Psoriasis (Pso) is a chronic, systemic T cell-mediated inflammatory condition of the skin and joints occurring in approximately 0.2–2% of the world population and notably more frequent in Caucasians (10). Pso is characterized by irritating or painful erythematous patches or plaques with silvery scales (10, 11). Like MS, the pathogenesis of Pso is determined by a genetic predisposition modulated by environmental triggers. Due to the fact this is a systemic inflammatory process, Pso patients are at higher risk of developing cardiovascular disease (12), cerebral vascular ischemia (13), chronic kidney disease (14), and uveitis (15). Given this wide range of associated co-morbidities, Pso has a significant negative physical and psychological impact on quality of life (16) and in severe Pso, shortened life expectancy (17, 18).

Like other immune-mediated conditions, MS patients exhibit a higher personal and familial rate of other autoimmune diseases (19). One case-control study in the US showed that Pso was the second most common co-existing autoimmune disease in MS patients after Hashimoto thyroiditis (20) but the association between Pso and MS is inconsistent with wide variance in reported incidence and prevalence (21, 22). The observed correlation between MS and Pso may be driven by shared genetic susceptibility (HLA alleles) as well as overlapping immunological pathways, particularly the IL-23/IL-17 axis and the generation of pro-inflammatory cytokines (IL-17 and TNF-α) (23, 24).

The current prevalence of Pso in patients with MS in Australia is unknown. We sought to determine the accurate frequency of Pso among patients with MS using a focused questionnaire in a prospective, clinic-based study in Sydney.

## Materials and methods

The study was conducted using a survey with 29 directed questions (Appendix 1) either distributed via email or performed face-to-face on MS patients aged 18 years and over attending a large MS specialist outpatient clinic at Royal North Shore Hospital (RNSH), Sydney, from July 2018 to December 2022, which supports 1142 patients. Two hundred and four consecutive MS patients were randomly selected and invited to participate. All participating patients provided their consent.

The study was approved by the Northern Sydney Local Health District Human Research Ethics Committee, which is constituted in accordance with the National Statement on Ethical Conduct in Human Research, 2007 (NHMRC; 2021/ETH00892).

In an interview setting, patients completed a questionnaire which aimed to determine the incidence and prevalence of Pso and to understand observations of dermatoses made in MS clinics. The survey on the platform “REDCap” was designed to capture demographic information, symptoms, and history of Pso. All the information was then extracted and entered into the REDCap database along with relevant information on the history of skin conditions, signs and symptoms of dermatosis. Association between categorical variables were tested using Chi-square or Fisher's exact test. Continuous variables were compared between participants with and without Pso groups using independent-samples *t*-tests for normally distributed data, and Mann–Whitney U test for non-normally distributed data. The comparison of incidence of skin symptoms between with and without Pso groups was conducted using binary logistic regression reporting odds ratio (OR) and the associated 95% confidence interval (CI).

## Results

A total of 204 patients were surveyed: 137 (67.2%) females and 67 (32.8%) males. The age of the patients ranged between 17 and 81 years with the mean age 48.8 years (SD = 13.6). The median Expanded Disability Status Scale (EDSS) score of the cohort was 1.5 (IQR: 0.75–2.50), with a disease duration of MS of 15 years (IQR: 10–21 years).

MS patients with Pso had a mean age of 50.8 years (SD: 13.7) and a median EDSS score of 1.5 (IQR: 0–3.9), with a disease duration of MS of 15 years (IQR: 9.25–19.50). In comparison, non-Pso MS patients had a mean age of 48.4 years (SD: 13.6) and a median EDSS score of 1.5 (IQR: 1.0–2.5), with a disease duration of MS of 15 years (IQR: 10–21; Table 1).

**Table 1:** Demographics and clinical characteristics of MS cohort.

**Characteristics**	**MS (*n =* 204)**	**MS without Pso (*n =* 176)**	**MS with Pso (*n =* 28)**	***p*-value**
**Age (years), mean (+/- SD)**	48.8 (±13.6)	48.4 (±13.6)	50.8 (±13.7)	0.40^+^
**Sex ratio (F:M)**	2.04:1 (67.2%: 32.8%)	1.9:1 (65.3%: 34.7%)	3.7:1 (78.6%: 21.4%)	0.120^++^
**EDSS, median (IQR)**	1.5 (1.0–2.5)	1.5 (1.0–2.5)	1.5 (0.0–3.9)	0.63^+++^
**Disease duration (years), median (IQR)**	15 (10–21)	15 (10–21)	15 (9.3–19.5)	0.9^+++^

^+^ based on independent samples t-test, ^++^based on Fisher's exact-test, ^+++^Mann-Whitney U test.

Due to loss to follow-up or transfer of care to another network, immunotherapy data is available for 196 out of 204 patients (Table 2). Of the total MS cohort, 57.2% were managed with B-cell therapies (ofatumumab and ocrelizumab). Among patients without Pso, 60.1% received B-cell therapies, whereas only 37% of those with Pso were treated with these agents. MS patients with Pso who receive B-cell therapy are more likely to be prescribed Kesimpta (ofatumumab) rather than Ocrevus (ocrelizumab) when compared to MS patients without Pso.

**Table 2:** Comparison of disease modifying therapy use in patients with and without Pso in the multiple sclerosis cohort.

**Disease Modifying Therapy**	**MS (*n =* 196) %**	**MS without Pso (*n =* 168) %**	**MS with Pso (*n =* 28) %**
**Nil**	4.1	2.4	14.3
**Ocrelizumab**	40.0	45.2	10.7
**Ofatumumab**	17.9	16.1	28.6
**Natalizumab**	17.4	17.2	17.9
**Dimethyl fumarate**	4.1	4.8	0
**Teriflunomide**	2.6	1.8	7.1
**BRACE**	0.5	0.6	3.6
**Alemtuzumab**	5.6	5.4	7.1
**Siponimod**	2.1	1.8	0
**Glatiramer acetate**	0.5	0.6	0
**Autologous stem cell transplant**	1.0	0.6	3.6
**Cladribine**	2.1	1.2	7.1
**Tolebrutinib**	0.5	0.6	0

The observed frequency of Pso in MS patients was 13.7% (28/204; 95% CI: 9.63–19.20%) as compared to an estimated 2.4% lifetime prevalence rate of physician-diagnosed Pso in the general Australian adult population (25).

In our study, of the 176 participants without a Pso diagnosis, various skin symptoms were reported including 6-month-history of intermittent irritation or erythematous rash (68/176, 38.6%), seasonal skin changes (69/176, 39.2%) and flaky, peeling, or scaly skin (35/176, 19.9%). Moreover, 54/176, 30.7% experienced dandruff; 21/176, 11.9% had thickened scaly skin behind ears and scalp; 25/176, 14.2% reported nail changes; and 24/176, 13.6% reported a family history of Pso. Interestingly, 9/28 or 32.1% of MS patients with Pso reported a concurrent diagnosis of eczema Table 3. Furthermore, except for new skin manifestations and dandruff, MS with Pso reported significantly higher odds of all other skin symptoms as compared with those without Pso (Odds ratio ranging between 2.01 and 22.14).

**Table 3:** Skin manifestations in MS patients.

**Skin manifestations**	**MS (*n =* 204)**	**MS without Pso (*n =* 176)**	**MS with Pso (*n =* 28)**	**OR (95% CI)**	***p*-value**
**New skin manifestations since a diagnosis of MS**	66 (32.4%)	53 (30.1%)	13 (46.4%)	2.01 (0.90–4.52)	0.09
**6-month-history of intermittent pruritus or erythematous rash**	176 (86.3%)	68 (38.6%)	22 (78.6%)	5.82 (2.25–15.09)	< 0.001
**Seasonal skin changes**	82 (40.2%)	69 (39.2%)	13 (46.4%)	2.93 (1.30–6.62)	0.01
**Thickened scaly skin of extensors, behind ears or scalp**	42 (20.6%)	21 (11.9%)	21 (75.0%)	22.14 (8.40–52.40)	< 0.001
**Flaky, peeling or scaly of skin**	55 (27.0%)	35 (19.9%)	20 (71.4%)	10.07 (4.10–24.76)	< 0.001
**Dandruff**	63 (30.9%)	54 (30.7%)	9 (32.1%)	1.07 (0.45–2.52)	0.876
**Nail changes**	34 (16.7%)	25 (14.2%)	9 (32.1%)	2.86 (1.16–7.03)	0.022
**Diagnosis of eczema**	28 (13.7%)	19 (10.8%)	9 (32.1%)	3.91 (1.55–9.87)	0.004

## Discussion

This study demonstrates that the frequency of Pso in MS patients treated in a tertiary clinic is six times greater (13.7% vs. 2.4%) than the Australian population (25). Previous studies have reported rates of between 0.4 and 7.7% (21, 22) and epidemiological data has consistently shown rates of Pso are high in MS.

In our study, MS patients with concurrent Pso showed a significantly higher frequency of skin symptoms, particularly those characteristics of Pso, including thickened scaly skin (OR: 22.14, *p* < 0.001) and flaky or peeling skin (OR: 10.07, *p* < 0.001). Intermittent rash or pruritus, seasonal skin changes, nail changes, and eczema were also significantly more common. Although new skin manifestations since MS diagnosis showed a non-significant trend (OR: 2.01, *p* = 0.09), the overall pattern supports the need for increased dermatological surveillance among MS patients.

While we observed differences in disease modifying therapy (DMT) use between patients with and without Pso, we cannot definitively conclude that Pso influenced DMT selection, as the timing of Pso onset relative to DMT initiation was not captured. This represents an important limitation of our analysis and should be interpreted with caution. Additional limitations include incomplete immunotherapy data for three patients due to loss of follow-up or transfer of care to different healthcare networks.

Interestingly, ocrelizumab use was lower among patients with Pso, which may reflect rising clinical awareness of Pso as a potential complication associated with this therapy. In contrast, ofatumumab, a more recently introduced anti-CD20 agent (approved in 2021, compared to 2018 for ocrelizumab) was more frequently used in the Pso group. This may be due to its more recent introduction and the relative lack of reported Pso cases at the time of treatment. The precise mechanism by which anti-CD20 therapy may trigger psoriasis in MS patients remains unclear. However, depletion of B cells may lead to dysregulation of T helper subsets, particularly Th1 and Th17, while the suppression of IL10-producing regulatory B cells (Bregs) may impair peripheral tolerance, thereby promoting psoriatic inflammation (26, 27).

On the other hand, cladribine and teriflunomide appeared to be more commonly used in patients with Pso, possibly reflecting the notion of the treating clinicians that these therapeutics are beneficial for psoriasis as well. Behrens et al. demonstrated that leflunomide was effective and safe in 514 European patients with psoriatic arthritis and another small study showed the efficacy of cladribine in psoriatic arthritis (28, 29).

Cendroswki and colleagues were the first to report an association in 1989 (30), and was followed by several population-based studies that reported a positive correlation between Pso and MS. A large single center, retrospective, cross-sectional study of 5,097 Pso patients and 1,829 MS patients in the US, found a significant association (OR = 1.521; 95% CI 1.01–2.29; *P* = 0.04) between the two conditions despite adjusting for potential confounders (31). A retrospective case-control study of 3,456 MS patients analyzing the association between Pso and MS disease activity and progression showed that 1.3% of patients with MS have Pso. Among these, 78% had Pso prior to MS diagnosis and the presence of Pso prior to the diagnosis of MS predicted a milder disease course and a longer interval to the second relapse (32). Three other small case-control studies reported higher frequencies of Pso in MS patients (33–35).

The presence of Pso prior to a diagnosis of MS has proven to be a risk factor for the disease. A Danish nationwide cohort study investigated the probability of developing MS in patients with mild (*n* = 58,628) or severe (*n* = 9,952) Pso and demonstrated significantly increased rates of MS in both groups compared to the reference population (36). The incidence of MS was highest among patients with severe Pso suggesting a disease severity-dependent increased risk of MS in Pso patients {mild Pso [incidence rate ratio (IRR), 1.84; 95% CI, 1.46–2.30] and severe Pso (IRR, 2.61; 95% CI, 1.44–4.74)} (36). Summary data including systematic reviews and meta-analyses support the association (11, 37). Dobson et al. found an overall increased risk for Pso in MS patients (OR = 1.31, 95% CI 1.09–1.57, *P* < 0.0001) (37). Interestingly, in this study the sex ratio of MS patients with Pso closely mirrored the known MS sex ratio (usually 3:1) rather than that of Pso (usually 1:1) (8, 38). This may also suggest shared immunogenetic pathogenesis among the two diseases and perhaps a particular MS/Pso phenotype.

The concomitance of Pso and MS may reflect shared genetic and environmental factors resulting in dysregulation of immune function. The prevalence of Pso and MS varies between geographical regions with both exhibiting a latitudinal gradient (greater prevalence toward the poles) and this has been shown to be partly explained by sun exposure and vitamin D levels (10, 39, 40). Additionally, infections along with lifestyle factors including smoking (both passive and active), diet, body mass index and stress have been proposed to impact the onset and progression of both diseases (41).

Pso and MS share other characteristics including heritability. Allelic variation in HLA-DRB1 and IL-12B are present in both diseases and genome wide screens reveal cross-over of many of the small contributory allelic variants that exert suspected effects on immune regulatory genes (11). Although the exact immunogenetic pathways for both diseases are unclear, activation of Th-1 and Th17 cells has been observed in both MS and Pso. In MS, inflammatory cytokines including interferon gamma; tumor necrosis factor alpha; and interleukins (IL)-17, 21, 22, and 26 have been implicated as primary pathogenic factors (42). These cytokines also play a key role in Pso (11).

IL-17 has been shown to be implicated in the pathogenesis of both Pso and MS (43). The immunologic role of IL-17 in Pso has been well established including IL-17 associated genes and an upregulation of IL-17A (44). IL-17A-targeting antibodies have been effectively prescribed for the treatment of Pso and yield promising outcomes in attenuating MS progression in an early-stage clinical trial (45). A high level of IL-17 was observed in perivascular cells isolated in the areas of active MS lesions suggesting the potential role of IL-17 in early stages of disease (46). This finding was in line with an animal study showing impressive clinical efficacy when anti-IL-17A was given to mice at the induction of experimental autoimmune encephalomyelitis (EAE). By contrast, mice lacking IL-17A were resistant to MS (47).

However, the immunological variance of these diseases cannot be over-simplified, and this is exemplified by the finding that some therapeutics will treat both diseases, whilst others will exacerbate the concomitant condition. Dimethyl fumarate is effective treatment for Pso and MS at least partly by downregulating the function of Th1 and Th17 T-lymphocytes (48, 49). Conversely, B-lymphocyte depletion and interferon beta treatment for MS can lead to exacerbation of Pso, while TNF-alpha blockade via monoclonal antibodies against the cytokine or it's receptor for psoriatic arthritis can exacerbate MS or induce *de novo* CNS demyelination (22, 50–52).

We are uncertain why the frequency of Pso is so high in our cohort and would expect similar rates among Caucasian populations. It may relate to data collection and the directed analysis of patients with questionnaires rather than based on population-based registries which may be prone to under-reporting or reduced data-capture. Alternatively, due to the unblinded nature of this observational cohort, the reporting from this series may be accentuated. Collectively, the rate of Pso is higher in MS patients, and this warrants further investigation to unearth common pathogenic mechanisms that may lead to better understanding of both diseases and precision treatments.

## Data Availability

The original contributions presented in the study are included in the article/Supplementary material, further inquiries can be directed to the corresponding author.
