# Supplementary material for: The frequency of psoriasis in Australian multiple sclerosis patients
Source: Front Neurol. 2025 Oct 2;16:1663015. doi: 10.3389/fneur.2025.1663015 (PMC12527903; doi:10.3389/fneur.2025.1663015)
Supplement: Supplementary file 1 [file Data_Sheet_1.pdf]

Appendix 1: Survey questionnaires

Psoriasis in Patients with Multiple Sclerosis

Name ________________________ DOB ___________________________

1. **Have you had an itchy <u>or</u> red rash, which was coming and going for at least 6 months?**
   a) Yes
   b) No

2. **At what age did this itchy rash first occur?**

   a) 10 years old or younger
   b) 11 - 30 years old
   c) 31 – 40 years old
   d) 41 – 50 years old
   e) 51 – 60 years old
   f) 61 years old or older

3. **In the last year, have you suffered from a dry skin in general?**
   a) Yes
   b) No

4. **Has this itchy or red rash at any time affected any of the following places: the folds of the elbows, behind the knees, under the buttocks or around the neck, ears, or eyes?**
   a) Yes
   b) No

5. **Have you noticed that your skin is itchier in the summer and drier in the winter?**
   a) Yes
   b) No

6. **Is your skin worse in winter and better in the summer?**
   a) Yes
   b) No

7. **Have you ever had thickened scaly skin which affects elbows, knees, behind your ears or scalp?**
   a) Yes
   b) No

8. **Have you noticed that your skin is frequently flaky, peeling or scaly?**
   a) Yes
   b) No

9. **Have you noticed nail changes such as small dents in your nail, or yellow discolouration of the nail bed?**
   a) Yes
   b) No

10. **Do you get dandruff?**
    a) Yes
    b) No

11. **Do you have a history of diagnosed eczema?**
    a) Yes

b) No

12. **Do you have a history of diagnosed psoriasis?**
    a) Yes
    b) No

13. **Does anyone in your family have eczema?**

    a) Yes
    b) No

14. **Does anyone in your family have psoriasis?**
    a) Yes
    b) No

15. **Have you been diagnosed with any other skin conditions?**
    Free text answer

16. **Do your joints hurt?**
    a) Yes
    b) No

17. **Have you taken anti-inflammatory more than twice a week for joint pain in the last 3 months?**
    a) Yes
    b) No

18. **Do you wake up at night because of lower back pain?**
    a) Yes
    b) No

19. **Do you feel stiffness in your hands for more than 30 minutes in the morning?**
    a) Yes
    b) No

20. **Do your wrists and fingers hurt?**
    a) Yes
    b) No

21. **Do your wrists and fingers swell?**
    a) Yes
    b) No

22. **Does one finger hurt and swell for more than 3 days?**
    a) Yes
    b) No

23. **Does your Achilles tendon swell?**
    a) Yes
    b) No

24. **Do your feet or ankles hurt?**
    a) Yes
    b) No

25. **Do your elbows or hips hurt?**
    a) Yes

b) No

26. Have you had lower back pain and stiffness for more than 3 months, which improves with exercise, but is not relieved by rest?

a) Yes
b) No

27. Do you have a diagnosis of any of the following forms of arthritis (select all that apply)

a) Ankylosing spondylitis
b) Reactive arthritis
c) Rheumatoid arthritis
d) Enteropathic arthritis (joint disease associated with inflammatory bowel disease)
e) Psoriatic arthritis

28. Do you have a diagnosis of Behcet's disease?

a) Yes
b) No

29. Have you developed new skin symptoms since being diagnosed with multiple sclerosis?

a) Yes
b) No

END OF SURVEY
